# The choice that matters: the relative influence of socioeconomic status indicators on chronic back pain- a longitudinal study

**DOI:** 10.1186/s12913-017-2735-9

**Published:** 2017-12-02

**Authors:** Michael Fliesser, Jessie De Witt Huberts, Pia-Maria Wippert

**Affiliations:** Sociology of Health and Physical Activity, University of Potsdam, Am Neuen Palais 10, 14469 Potsdam, Germany

**Keywords:** Socioeconomic status, Indicators of socioeconomic status, Health inequality, Education, Job position, Income, Chronic back pain

## Abstract

**Background:**

In health research, indicators of socioeconomic status (SES) are often used interchangeably and often lack theoretical foundation. This makes it difficult to compare results from different studies and to explore the relationship between SES and health outcomes. To aid researchers in choosing appropriate indicators of SES, this article proposes and tests a theory-based selection of SES indicators using chronic back pain as a health outcome.

**Methods:**

Strength of relationship predictions were made using Brunner & Marmot’s model of ‘social determinants of health’. Subsequently, a longitudinal study was conducted with 66 patients receiving in-patient treatment for chronic back pain. Sociodemographic variables, four SES indicators (education, job position, income, multidimensional index) and back pain intensity and disability were obtained at baseline. Both pain dimensions were assessed again 6 months later. Using linear regression, the predictive strength of each SES indicator on pain intensity and disability was estimated and compared to the theory based prediction.

**Results:**

Chronic back pain intensity was best predicted by the multidimensional index *(beta* = 0.31, *p* < 0.05), followed by job position (*beta* = 0.29, *p* < 0.05) and education (*beta* = −0.29, *p* < 0.05); whereas, income exerted no significant influence. Back pain disability was predicted strongest by education (*beta* = −0.30, *p* < 0.05) and job position (*beta* = 0.29, *p* < 0.05). Here, multidimensional index and income had no significant influence.

**Conclusions:**

The choice of SES indicators influences predictive power on both back pain dimensions, suggesting SES predictors cannot be used interchangeably. Therefore, researchers should carefully consider prior to each study which SES indicator to use. The introduced framework can be valuable in supporting this decision because it allows for a stable prediction of SES indicator influence and their hierarchy on a specific health outcomes.

## Background

Economic inequality is increasing in many countries [1] and is associated with a variety of negative health outcomes [2, 3]. For this, socioeconomic inequality remains an important focus in health research. Despite the attention it has been afforded, the causal pathways leading from socioeconomic status (SES) to certain health outcomes are not fully understood yet [4]. One of the reasons for this may be that studies investigating the link between SES and health utilize different operationalizations to indicate a person’s SES. Common operationalizations are for example education (with higher educational attainment indicating higher SES), job position (with more prestigious positions or positions with more resources indicating higher SES) and income (higher income indicating higher SES) or a combination of these variables (usually by adding up scores for every single dimension) [5–8]. Although all these operationalizations are valid, they rely on different assumptions about the link between SES and health outcomes [8, 9]. As a result, the predicted influence of SES on specific health outcomes may vary depending on the used indicator, making it difficult to establish the links between SES and health. To illustrate this, several studies have already observed the change of the association between different SES measures and mortality and different associations with mortality have been found for each of these indicators: Geyer and colleagues found medium strong effects of income, small effects for education and very small effects for job position [10]. Duncan and colleagues found medium effects for family wealth and income, but no effects for education or job position [11]. This stresses the difficulty to talk about SES as a uniform predictor.

While these studies indicate that the relationship between SES and health depends on the selected SES indicator and therefore different indicators cannot be used interchangeably, they do not provide a rationale for selecting the relevant SES indicators to explain relationship between SES and certain health outcomes. This makes it difficult for researchers to select the relevant indicator(s) for their research question. Therefore, a theory-based framework is needed to help researchers to systematically select the indicator most suitable for their research question.

**Fig. 1:**
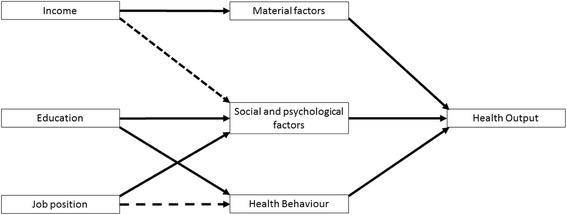
Pathways connecting SES indicators with health outputs (based on Social Determinants of Health by Brunner & Marmot, [4])

Such framework, which connects different aspects of social structure with health outcomes is suggested for example by Brunner and Marmot [4]. This model provides a general theory about the relationship between social structure and health and therefore can be applied to a wide range of more concrete research questions. It postulates that there are three main pathways linking social structure to health outcomes: Firstly, social structure shapes *material conditions* (e.g. pollution load, noise exposure), which have positive or negative effects on health. Secondly, social structure influences *social and psychological factors* (e.g. stress at work and at home, ability to cope with varying situations, vulnerability for anxiety and depression), which then influences health outcomes. Thirdly, social structure also has an impact on *health behaviour* (e.g. physical activity, dietary habits) which also influences well-being. Prior research shows, that different SES indicators (as manifestation of social structure) do not influence these three pathways to the same degree. Income is strongly associated with material factors as people with a higher income generally have for example more favourable living conditions [12], and to a small degree with social and psychological factors [9] and with health behaviour [13]. So, it can be assumed that out of the three possible pathways, income is most strongly associated with material factors and, to a smaller extent, with social and psychological factors and health behaviour. The second socioeconomic indicator, education, is associated with *social and psychological factors* [9, 14], and *health behaviour* [15, 16]. No clear evidence linking education and material factors has been found in previous research [17]. Therefore, it is assumed that education is associated with social and psychological factors and health behaviour, but not with the material environment. Job position is associated with *social and psychological factors*, both directly and mediated via working conditions [9, 18]. Additionally, to a smaller extent, job position is also associated with *health behaviour* [19, 20]. Based on this, it appears that while all SES indicators are connected to social and psychological factors, comparatively, income is most strongly linked with the material environment and education is most strongly linked with health behaviour (Fig. 1).

This model represents a very simplified representation of reality by not taking into account the fact that there is a correlation between the three different SES-indicators. For example, people having higher position in one SES domain often tend to have higher positions in other SES-domains [8]. Likewise, the mediating factors are also likely to be correlated, e.g. social and psychological factors such as high stress can lead to a worse health behaviour such as more smoking [21]. However, the main purpose of the model is to enable researchers to estimate a priori the impact of a certain SES indicators on a given health outcome, and therefore we believe that a simplified model is more useful as a decision-making tool for the selection of SES indicators in research. If it is plausible that for example mainly health behaviour influences a certain health outcome, then it could be estimated that the SES indicators most strongly associated with health behaviour (education and to a lesser extent job position) have a higher impact on the health outcome than indicators that are not associated with health behaviour (e.g. income) and therefore may be a better indicator for the chosen research question. In the present study it is analysed, if this selection framework is able to correctly predict the strength of the influence of different indicators on the development of chronic back pain and therefore may support researchers in choosing the appropriate indicator for their research question.

## SES and back pain

Acute and chronic back pain is an illness with a high lifetime prevalence [22], and a multifactorial aetiology [23, 24]. It is unequally distributed across different societal groups [25] and factors associated with the SES may play an important role in the development of back pain. Furthermore to date it has not been researched in this context. Therefore, chronic back pain is a suitable health domain to test the relative influence of different SES indicators. In scientific studies back pain often is assessed on two dimensions: pain intensity and disability caused by back pain [26]. Using the theoretical framework by Brunner and Marmot to select adequate SES predictor for chronic back pain, the following hypotheses about the link between SES and chronic back pain can be made.

**Fig. 2:**
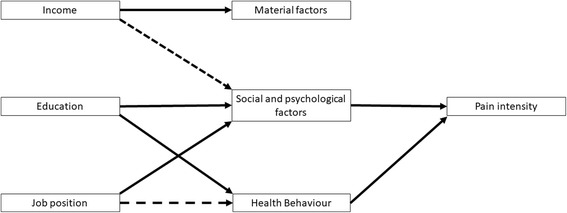
Pathways connecting SES indicators with Pain Intensity (based on Social Determinants of Health by Brunner & Marmot, [4])

Scholich and colleagues and Shaw, Pransky and Main indicated that social and psychological factors (stress, anxiety, depression, psychosocial working conditions) play an important role in back pain intensity [27, 28]. Furthermore health behaviour (especially physical activity) has been identified as influential for pain intensity [29]. Factors related to the material environment in the model by Brunner and Marmot, such as noise or air pollution on the other hand have, to our knowledge, not been found to be associated with development of chronic pain intensity. Using these insights, following assumptions are assumed: The single indicators education (influencing social and psychological factors and health behaviour) should be the strongest predictor on back pain intensity, followed by job position (influencing social and psychological factors and to a smaller extent health behaviour), whereas income (covering mainly material factors which play a minor role for chronic back pain) should have little influence (Fig. 2). The multidimensional index (created as combination of the three named single indicators) should be able to predict development of back pain intensity even better than the single indicators, since it combines the influence of the single indicators.

**Fig. 3:**
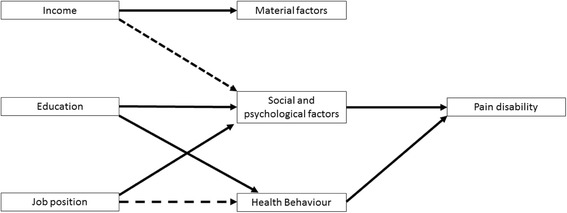
Pathways connecting SES indicators with Pain Disability (based on Social Determinants of Health by Brunner & Marmot, [4])

Chronic back pain disability is also associated with social and psychological factors [28, 30] and especially with health behaviour such as physical activity [29]. Material factors are again not clearly connected to disability. This is why we again expect education as the most influential (single) indicator, followed by job position and income (Fig. 3). The multidimensional index should also allow good predictions.

## Methods

### Sample

*N* = 145 patients who were in rehabilitation treatment because of chronic back pain (Mean age: 48.5, *SD*: 6.4; *Min*: 35, *Max*.: 60, 72 men). The recruitment was guided by the following criteria: 1) age between 35 and 60 2) in treatment because of disc prolapses (slipped disc) or spinal canal stenosis (narrowing of the spinal canal) 3) disease duration lasting more than 3 months. Patients with a recurrent operation were excluded. *N* = 107 patients participated in the follow up measurement after an average of 6.6 months (Dropout: 26%). Participants who failed to complete all relevant questions for the SES operationalisations education, job position, income and WS-index, were excluded leading to a final sample of 66 (Mean age: 48.2, *SD*: 5.9; *Min:* 36, *Max*: 60, 33 women). Before enrolling in the study, participants were informed about the study procedures and were required to sign a consent form. The survey respected the agreement of the Declaration of Helsinki and was approved by the independent Ethics Committee of the University of Potsdam (committee’s reference number 01/2012).

### Testing procedure

Initial measurements were performed in two rehabilitation clinics of Berlin/Brandenburg (Germany) where patients were enrolled for a 3 week rehabilitation program. After standardized medical examination by a physician, the participants filled out a questionnaire assessing the predictor indicators (sociodemographic and SES variables) as well as pain intensity and disability (t1). After leaving the clinic the participants rated after an average of 6.6 months again their pain intensity and disability via emailed or mailed questionnaires (t2).

### Instruments and data pre-processing

*Education* was assessed using the eight category classification of the International Standard Classification of Education (ISCED), combining school and vocational education (1 = primary education, 8 = university degree) [31, 32].

*Job position* was measured using the nine categories of the International Standard Classification of Occupation (ISCO-08), which pools jobs according to main tasks, skill level and specialisation (1 = managers, 9 = elementary occupations) [33].

*Net Household income* was asked through an open question according to the guidelines for sociodemographic standards [34] Then *household equivalent income* was computed by multiplying overall household income with weighting factors according to household members using OECD-modified scale suggestions. This scale assigns a value of 1 to the first person in a household, 0.5 to each additional adult and 0.3 to each child under 12 [35].

#### Winkler-Scheuch-index (WS-index)

Furthermore, a multidimensional socioeconomic variable was calculated. This index is recommended by the German working group for social epidemiology and used in multiple nationwide studies [36]. It is based on the three dimensions education, job position and household equivalent income. Each person gets a value between 1 and 7 for each of the single indicators. The total of these three values then determines the person’s score in the WS-index, resulting in an index from 3 (lowest score) to 21 [36].

*Chronic Pain intensity* (CPI) of back pain was evaluated for both t1 and t2 using the Chronic Pain Grade questionnaire (CPG) created by von Korff and colleagues [37]. This well-established and validated instrument [38] consists of three questions about the actual and the average intensity of back pain people experienced in the last 3 month as well as the worst experienced back pain in the last 3 months. Answering options ranged from 0 (no pain) to 10 (worst possible pain). After data transformation the mean for pain intensity ranges from 0 to 100 for each patient [37]. Internal consistency was satisfying for t1 (Cronbach’s Alpha = 0.66) and good for the t2 (Cronbach’s Alpha = 0.91).

*Subjective Pain disability (DISS)* was evaluated using three questions of the CPG questionnaire asking about how much the pain interfered with daily activities, recreational and social activities and with work (again with a final score of 0 to 100, [37]). Internal consistency was good for both measurement points (Cronbach’s Alpha t1 = 0.91; t2 = 0.95).

### Statistical analysis

After descriptively describing baseline data, four independent hierarchical regression analyses were conducted for each outcome (pain intensity and pain disability) using either education, job position, household equivalent income or the WS-index while controlling for age, sex and pain intensity at baseline in each model. As most studies only use a single indicator to represent SES, a separate model for each of the indicators was used as this allowed us to estimate how much the influence of the different indicators may vary if the other indicators are not taken into account.

Requirements of the regression analysis were tested with collinearity diagnosis, Durbin-Watson test and Kolmogorov Smirnoff test for normality of residuals. All analyses were performed with IBM SPSS Statistics 21.

## Results

Distribution of education and job position is presented in Table 1. The sample consisted mainly of people with middle to high education and job position. Distribution of age, income, the multidimensional index and the pain variables are presented in Table 2. The sample consists of people with average income and average WSI. Chronic pain intensity was on average 59 out of 100 points at the initial measurement (*SD* = 15.5) and dropped to 44 6 months later (*SD* = 25.0). Average limitation in everyday life was reported to be 34 out of 100 points at follow-up (*SD* = 27.7).

**Table 1:** Characteristics of study sample, categorical variables: (*N* = 92)

Educational degree	Percentage	Job position	Percentage
No educational degree	4.3	Managers	22.8
Primary education	5.4	Professionals	2.2
General secondary education	16.3	Technicians	21.7
Professional secondary education	56.5	Clerical Support Workers	7.6
Technical secondary education	4.3	Services and Sales Workers	13.2
Technical college degree	4.4	Skilled Agricultural Workers	1.1
University degree	8.7	Craft Workers	21.6
		Machine Operators	7.6
		Elementary Occupations	2.2

**Table 2:** Characteristics of study sample, constant variables

Variable	N	M	SD	Min.	Max.
Age	92	48.3	6.0	36	60
Income	92	1477	860	476	4666
WS-index	92	10.9	3.3	5.3	18.7
CPG pain intensity baseline	91	58.7	15.5	10	90
CPG pain intensity follow up	66	43.9	25.0	0.0	93.3
CPG disability follow up	66	33.7	27.7	0	96.7

The people who dropped out differed from the included sample regarding income (significantly higher in included sample). There were no significant differences between the groups regarding age, education and job position (tests performed with Mann-Whitney U-tests).

### SES composition and pain intensity

Beginning with the analysis of SES indicators on CPG pain intensity (Table 3), the WS-index showed the strongest influence *(beta* = −0.31), followed by job position *(beta* = 0.29) and education *(beta* = −0.29): People with higher overall SES, better job positions and better education indicated less pain in the past 3 months. All three associations had small effect sizes. Income had no significant influence on pain intensity. In general, the models, controlling for age, sex and baseline pain intensity, were able to explain between 22% (income) and 29% (WS-index) of the observed variance.

**Table 3:** Hierarchical regression models predicting influence of different operationalisations of SES on CPG pain intensity score (higher values more pain), controlled for age, sex and baseline pain (*N* = 66)

Model	SES indicator	R^2^	ΔR^2^	Beta	T-value	p
1	Education	.277	.078*	−.292	−2.56	.013*
2	Job	.262	.062*	.293	2.27	.027*
3	Income	.218	.019	−.140	−1.20	.234
4	WS-index	.290	.090*	−.310	−2.78	.007*

* = p < 0.05

### SES composition and disability

For CPG pain disability the strongest influence were exerted by education (*beta* = −0.30) and job position (*beta* = 0.29) (Table 4). Better educated people and with better job positions, on average, suffered less from disability because of pain. The effect sizes were small. Both the combined SES index and income had no significant influence. The models were able to explain between 19% (income) and 26% (education) of observed variance.

**Table 4:** Hierarchical regression models predicting influence of different operationalisations of SES on CPG disability (higher values, more disability), controlled for age, sex and baseline pain (N = 66)

Model	SES-indicator	R^2^	ΔR^2^	Beta	T-value	*p*
1	Education	.264	.079*	−.295	−2.56	.013*
2	Job	.243	.059*	.285	2.17	.033*
3	Income	.186	.002	−.047	−0.40	.692
4	WS-index	.232	.048	−.226	−1.95	.056

* = p < 0.05

## Discussion

This study first of all examined if the predicted influence of SES on the development of chronic back pain depends on the operationalization of the SES. The results show that the type of the SES indicator is of crucial importance and different indicators should not be used interchangeably, as already stressed out by other authors [8, 10]. In this case, the choice for income as the only SES predictor when investigating the influence of SES on chronic back pain would have led to the assumption, that there is no influence. This indicates the importance of a careful selection of SES indicators.

In order to select the most adequate SES indicator(s), a theory-based selection of SES indicators, applicable for different health domains, was proposed and tested. Using the suggested framework, it was expected that for back pain intensity the combined SES score would exert the strongest influence, followed by a weaker influence of education and job position and little or no influence of back pain intensity. Indeed, our findings indicated that the WS-index exerted the strongest influence. But our data show that, other than assumed, job position and education had an equally strong influence (indicated by the similar beta-values). Finally, we expected income to have the least predictive power and our data indicate that income was not associated with back pain intensity at all, although this may be due to the sample being too small to reveal the small effect of income.

With regard to our second health outcome, pain disability, it was hypothesized that the combined SES score would be the strongest predictor, followed by education and job position and to an even lesser extent income. The results reveal that, education was the strongest predictor, followed by job position and the combined SES-score.

Although the suggested framework was able to identify indicators and to differentiate them about their influence on chronic back pain, there are some arguments to respect: First of all the prediction assumed that job position would have considerably less influence than education in both models. This was not the case. This may be explained by the fact that the model is a simplified version of reality and assumes that the pathways leading from the SES indicators to the health outcome are independent, which is unlikely to be true in reality. Heikkila and colleagues showed for example that social and psychological factors influence health behaviour [39]. This makes it difficult to disentangle the pure influence of either psychosocial conditions or health behaviour and increases the difficulty to assess correctly how much stronger one of the indicators which influences either psychological and social factors or health behaviour is in comparison to the other. Nevertheless, the results indicate that, whenever health behaviour is expected to have a strong influence on a certain health outcome, then education could be expected to be more influential than job position, although the difference might not be very big.

The second unexpected result was that the WS-index has a not much stronger influence (as for back pain intensity) or even a less strong influence (as for back pain disability) than the single indicators. So the influence of the single indicators, other than assumed, do not add up to a stronger effect when they are combined. This is something Geyer already showed for acute back pain [25]. Following his considerations, we assume that the combined SES indicator will especially fail to exert more influence, if the influence of the single indicators is remarkably different, as it is the case especially for pain disability. So the combined score can only to be expected to be considerable more influential than the single indicators, if the single indicators are assumed to exert similar strong influence.

As a third result, we can also derive some conclusions for future treatment of chronic back pain patients: As we were able to show, out of the SES indicators in question job position and even stronger education had significant influence on the healing process in patients with chronic back pain. This means that people with lower job positions and less education have a higher risk for prolonged pain. Interventions should therefore focus especially on this group of people.

The results presented in this paper are afflicted by some limitations, namely the specificity of the observed sample (back pain patients after treatment in rehabilitation) and the relatively small number of participants, caused by the research design (6 month gap between first and second measurement point, exclusion of all people who did not complete all SES questions). Although the excluded and included people did not differ significantly regarding age, education and job position, they did differ in income (with lower income in the drop outs) and possibly in other variables not observed. This could influence the results, especially the association between income and low back pain. It could therefore be that the strength of the relationship may be underestimated because of the higher drop-out of people with low income.

A repetition of the research design with a more heterogeneous and larger sample would make the results more reliable and would allow to use more complex statistical methods like structural equitation modelling. Furthermore the suggested framework was tested in only one health domain. It is therefore not certain if the framework will also work in other domains (although this is expected).

## Conclusion

We were able to show, that theoretical modelling in the suggested way can be a useful tool in the selection of SES indicators. We recommend that researchers use such approaches to decide on a more informed basis which indicator to choose for their research questions. This may help to explore the relationship between SES and health outcomes in more detail and reduces the risk of overlooking connections between SES and a health domain because of inappropriate SES selection.

## Abbreviations

CPG: Chronic pain grade questionnaire
CPI: Chronic pain intensity
DISS: Subjective pain disability
SES: Socioeconomic status
WS-Index: Winkler-Scheuch-Index

## References

[1] OECD (2011). An overview of growing income inequalities in OECD countries: main findings.

[2] Pickett KE, Wilkinson RG (2015). Income inequality and health: a causal review. Soc Sci Med.

[3] Lampert T, Richter M, Schneider S, Spallek J, Dragano N. Soziale Ungleichheit und Gesundheit: Stand und Perspektiven der sozialepidemiologischen Forschung in Deutschland. Bundesgesundheitsblatt Gesundheitsforschung Gesundheitsschutz. 2015; 10.1007/s00103-015-2275-6. 10.1007/s00103-015-2275-6 26631008

[4] Brunner G, Marmot MG, Marmot MG, Wilkinson RG (2011). Social organisation, stress, and health. Social determinants of health.

[5] Adler NE, Conner Snibbe A (2003). The role of psychosocial processes in explaining the gradient between socioeconomic status and health. Current Directions in Psychol Sci.

[6] Fliesser M, Klipker K, Wippert P-M. Zur Verwendung des soziookonomischen Status in der Gesundheitsforschung am Beispiel Ruckenschmerz - systematisches Review. Gesundheitswesen. 2016; 10.1055/s-0042-112460. 10.1055/s-0042-112460 27756089

[7] Galobardes B, Shaw M, Lawlor DA, Lynch JW (2006). Indicators of socioeconomic position (part 2). J Epidemiol Community Health.

[8] Hradil S, Richter M, Hurrelmann K (2009). Was prägt das Krankheitsrisiko- Schicht, Lage, Lebensstil?. Gesundheitliche Ungleichheit: Grundlagen, Probleme, Perspektiven.

[9] Gallo LC, Matthews KA (2003). Understanding the association between socioeconomic status and physical health: do negative emotions play a role?. Psychol Bull.

[10] Geyer S, Hemström O, Peter R, Vågerö D (2006). Education, income, and occupational class cannot be used interchangeably in social epidemiology. Empirical evidence against a common practice. J Epidemiol Community Health.

[11] Duncan GJ, Daly MC, McDonough P, Williams DR (2002). Optimal indicators of socioeconomic status for Health Research. Am J Public Health.

[12] Skalicka V, van Lenthe F, Bambra C, Krokstad S, Mackenbach J (2009). Material, psychosocial, behavioural and biomedical factors in the explanation of relative socio-economic inequalities in mortality: evidence from the HUNT study. Int J Epidemiol.

[13] Campbell DJT, Ronksley PE, Manns BJ, Tonelli M, Sanmartin C, Weaver RG (2014). The association of income with health behavior change and disease monitoring among patients with chronic disease. PLoS One.

[14] Lipowicz A, Szklarska A, Malina RM (2014). Allostatic load and socioeconomic status in polish adult men. J Biosoc Sci.

[15] Jarvis MJ, Wardle J, Marmot MG, Wilkinson RG (2011). Social patterning of individual health behaviours: the case of cigarette smoking. Social determinants of health.

[16] Giskes K, Turrell G, van Lenthe FJ, Brug J, Mackenbach JP. A multilevel study of socio-economic inequalities in food choice behaviour and dietary intake among the Dutch population: the GLOBE study. Public Health Nutr. 2006; 10.1079/PHN2005758. 10.1079/phn2005758 16480537

[17] Bobak M, Hertzman C, Skodova Z, Marmot M (2000). Own education, current conditions, parental material circumstances, and risk of myocardial infarction in a former communist country. J Epidemiol Community Health.

[18] Wang J, Smailes E, Sareen J, Schmitz N, Fick G, Patten S (2012). Three job-related stress models and depression: a population-based study. Soc Psychiatry Psychiatr Epidemiol.

[19] Mäkinen T, Kestilä L, Borodulin K, Martelin T, Rahkonen O, Leino-Arjas P, Prättälä R (2010). Occupational class differences in leisure-time physical inactivity - contribution of past and current physical workload and other working conditions. Scand J Work Environ Health.

[20] Pampel FC, Krueger PM, Denney JT (2010). Socioeconomic disparities in health behaviors. Annu Rev Sociol.

[21] Ansell EB, Gu P, Tuit K, Sinha R (2012). Effects of cumulative stress and impulsivity on smoking status. Hum Psychopharmacol.

[22] Breivik H, Collett B, Ventafridda V, Cohen R, Gallacher D (2006). Survey of chronic pain in Europe: prevalence, impact on daily life, and treatment. Eur J Pain.

[23] Chou R, Shekelle P (2010). Will this patient develop persistent disabling low back pain?. JAMA.

[24] Manchikanti L, Singh V, Falco FJE, Benyamin RM, Hirsch JA (2014). Epidemiology of low back pain in adults. Neuromodulation.

[25] Geyer S (2008). Einzelindikator oder Index? Masse sozialer Differenzierung im Vergleich. Gesundheitswesen.

[26] von KM, Jensen MP, Karoly P (2000). Assessing global pain severity by self-report in clinical and health services research. Spine.

[27] Scholich SL, Hallner D, Wittenberg RH, Hasenbring MI, Rusu AC (2012). The relationship between pain, disability, quality of life and cognitive-behavioural factors in chronic back pain. Disabil Rehabil.

[28] Shaw WS, Pransky GS, Main CJ, Hasenbring MI, Rusu AC, Turk DC (2012). Work-related risk factors for transition to chronic back pain and disability. From acute to chronic back pain: risk factors, mechanisms, and clinical implications.

[29] Henchoz Y, Kai-Lik So A (2008). Exercise and nonspecific low back pain: a literature review. Joint Bone Spine.

[30] Alschuler KN, Theisen-Goodvich ME, Haig AJ, Geisser ME (2008). A comparison of the relationship between depression, perceived disability, and physical performance in persons with chronic pain. Eur J Pain.

[31] UNESCO (2012). International standard classification of education: ISCED 2011.

[32] Bohlinger S (2012). Internationale Standardklassifikation im Bildungswesen.

[33] Statistik Austria (2011). ISCO 08- gemeinsame deutschsprachige Titel und Erläuterungen.

[34] Hoffmeyer-Zlotnik JHP, Warner U, Baur N, Blasius J (2014). Soziodemographische Standards. Handbuch Methoden der empirischen Sozialforschung.

[35] OECD (2014). Adjusting household incomes: equivalence scales.

[36] Lampert T, Kroll LE, Müters S, Stolzenberg H (2013). Messung des sozioökonomischen Status in der Studie “Gesundheit in Deutschland aktuell” (GEDA). Bundesgesundheitsblatt Gesundheitsforschung Gesundheitsschutz.

[37] Korff M, Ormel J, Keefe FJ, Dworkin SF (1992). Grading the severity of chronic pain. Pain.

[38] Keller S, Bann CM, Dodd SL, Schein J, Mendoza TR, Cleeland CS (2004). Validity of the brief pain inventory for use in documenting the outcomes of patients with noncancer pain. Clin J Pain.

[39] Heikkila K, Fransson EI, Nyberg ST, Zins M, Westerlund H, Westerholm P (2013). Job strain and health-related lifestyle: findings from an individual-participant meta-analysis of 118,000 working adults. Am J Public Health.

